# *Staphylococcus aureus* and *Enterococcus faecium* isolated from pigeon droppings (*Columba livia*) in the external environment close to hospitals

**DOI:** 10.1590/0037-8682-0353-2021

**Published:** 2022-08-22

**Authors:** Henrique Vieira Gartz de Vasconcellos, Kerollyn Fernandes Bernardes Silva, Horácio Montenegro, Camila Botelho Miguel, Polyana Tizioto, Ferdinando Agostinho, Marcelo Costa Araújo, Rosineide Marques Ribas, Marcos Vinícius da Silva, Siomar de Castro Soares, Virmondes Rodrigues, Deivid William da Fonseca Batistão, Carlo José Freire Oliveira, Wellington Francisco Rodrigues

**Affiliations:** 1 Universidade Federal do Triângulo Mineiro, Instituto de Ciências Biológicas e Naturais, Laboratório de Imunologia e Bioinformática, Uberaba, MG, Brasil.; 2 NGS Soluções Genômicas, Piracicaba, SP, Brasil.; 3 Universidade Federal de Uberlândia, Instituto de Ciências Biomédicas, Laboratório de Microbiologia Molecular, Uberlândia, MG, Brasil.; 4 Universidade Federal de Uberlândia, Escola de Medicina, Uberlândia, MG, Brasil.

**Keywords:** Staphylococcus aureus, Enterococcus faecium, Columbidae, Feces, Drug Resistance

## Abstract

**Background:**

Domestic pigeons carry pathogens in their droppings, posing a potential public health problem.

**Methods:**

The phenotypic and genotypic antimicrobial resistances of *Staphylococcus aureus* and *Enterococcus faecium* in the feces of urban pigeons near hospitals with intensive care units were measured.

**Results:**

Twenty-nine samples showed Enterococcus growth, whereas one was positive for S. aureus. The *S. aureus* isolate was sensitive to the antibiotics tested via antibiogram, however resistance genes were identified. *E. faecium* isolates showed phenotypic resistance to gentamicin, erythromycin, and ciprofloxacin.

**Conclusions:**

Antimicrobial profiles harmful to health were demonstrated in bacterial pathogens isolated from the external environment of hospitals.

Domestic pigeons (*Columba livia* Gmelin, 1789) are the most common animal species in urban environments, where they can find abundant food sources, shelters, and do not have natural predators^1^. Pigeons are often reported as reservoirs of various zoonotic pathogens^2^ ^-^ ^5^; therefore, their presence near hospitals and other patient care settings can be dangerous because of the possibility of pathogen dissemination within the healthcare environment, especially among immunocompromised populations such as intensive care unit patients. 

Healthcare-associated infections are frequent adverse events, often resulting in prolonged hospitalization time, treatment failures, post-surgical and post-organ transplantation complications, psychological stress, increased mortality, and increased costs for healthcare systems^6^. Concerning the etiology of nosocomial infections, *Enterococcus* spp. and *Staphylococcus aureus* are the most frequently reported gram-positive organisms in epidemiological studies from recent decades. This is mainly due to the increasing prevalence of their antibiotic-resistant variants, such as methicillin-resistant *S. aureus* and vancomycin-resistant *Enterococcus* ^7^.

This study aimed to isolate and characterize the antimicrobial resistance profiles of *S. aureus* and *E. faecium* from pigeon droppings collected within the vicinity of hospitals equipped with intensive care units in Macrorregião Sul do Triângulo Mineiro, Brazil.

For 3 consecutive weeks, pigeon stool samples (n = 96) were obtained from the external areas of 4 hospitals in the south of Triângulo Mineiro (Minas Gerais, Brazil) between July and August 2018. For each facility, 8 weekly collections were made (alternating 4 dry and 4 fresh samples), at an interval of 48 h, avoiding repeated sampling from the same excreta. Samples were inoculated on Baird-Parker and Enterococcosel agar media (MBiolog Diagnosticos, Contagem, MG, Brazil), and cultured at 36.0 °C for 24-48 h. *Staphylococcus* spp. were identified by Gram staining, colony morphology, and catalase and coagulase tests. *Enterococci* were identified by Gram staining, 6.5% NaCl broth growth, bile esculin hydrolysis, L-pyrrolidonyl-β-naphthylamide hydrolysis (PYR), and motility tests (MBiolog Diagnosticos). Positive isolates identified from these tests were characterized using a commercial kit for *Enterococcus* identification (Probac, São Paulo, Brazil) following the manufacturer’s instructions. Viable cells were counted using the pour plate method, and the findings were expressed as colony-forming units (CFU/mL).

Antimicrobial susceptibility was measured on Müeller-Hinton agar (BD Biosciences, Franklin Lakes, NJ, USA) according to the guidelines of the Clinical and Laboratory Standard Institute (CLSI, 2017). The antimicrobial panel for *Enterococcus faecium* included ampicillin (10μg), imipenem (10μg), norfloxacin (10μg), vancomycin (30μg), tigecycline (15μg), linezolid (30 μg), nitrofurantoin (100μg), streptomycin (330 μg), and gentamicin (10 μg) to detect high levels of aminoglycoside resistance (HLR-A). For *Staphylococcus* spp., amikacin (30 μg), ampicillin (10 μg), cefepime (30 μg), ciprofloxacin (5 μg), clindamycin (2 μg), cephalothin (30 μg), chloramphenicol (30 μg), and ceftazidime (30 μg) were used. All antimicrobials were purchased from Laborclin (Paraná, Brazil).

DNA was extracted and purified using a QIAamp DNA Stool Mini Kit (Qiagen, Venlo, Netherlands). The amount of DNA in the samples was determined using a NanoDrop spectrophotometer.

Quality (integrity) analysis, quantification, library preparation, and sequencing were performed at the LaCTAD laboratory (Campinas, Brazil). Quality was measured using a Bioanalyzer 2100 (Agilent Technologies, Santa Clara, CA, USA). Absence of degradation peaks or dragging of degraded DNA was observed, and quantification of intact samples was determined using a Qubit 2.0 fluorometer (Thermo Fisher Scientific, Waltham, MA, USA). The genome library was prepared using the Nextera XT DNA Library Preparation Kit (Illumina, San Diego, CA, USA) following the manufacturer’s guidelines, and evaluated using Bioanalyzer. Then, these were quantified in Qubit, underwent quantitative PCR with the KAPA Fast Universal kit, and sequentially diluted to prepare the pools. Subsequently, flow-cell clustering was performed, followed by sequencing on HiSeq2500 (Illumina), with an expected coverage of approximately 10 million reads per library. Sequencing reads were processed using 3 tools from the BBTools version 38.79 package (https://sourceforge.net/projects/bbmap/, https://jgi.doe.gov/data-and-tools/bbtools/). Illumina adapters and low-quality bases were removed using BBDuk (k = 23; mink = 9; hdist = 1; ktrim = r tpe tbo; minlen = 135; qtrim = rl; trimq = 15; maq = 25). BBMerge was used to correct errors in overlapping regions between the forward and reverse reads (ecco = t; mix = t; strict = t), while Clumpify was used for another round of sequencing error correction. An assembly was then made with metaSPAdes version 3.14.1, using the parameters: k = 31, 51, 71, 91, and 111. Contigs smaller than 200 bp were removed. 

The genome draft underwent contaminant analysis using BlobTools pipeline (version 1.1.1). BlobTools detects contaminants by searching with BLAST + (version 2.10.0) and evaluating the coverage of the sequence map against the assembled genome draft. Contigs identified as contaminants were removed using BlobTools, and contamination of the final genome sketch was assessed using CheckM pipeline with an independent method. This method uses marker genes specific to a given genomic lineage within a reference phylogenomic tree to estimate genome completeness and contamination. CheckM uses Prodigal (version 2.6.3) to identify genes in the genome draft and obtain the amino acid sequence of the predicted genes, HMMER (version 3.2.1) to identify the families of the identified proteins, and pplacer (version 1.1. alpha19) to determine the positions of these proteins in a pre-calculated phylogenetic tree. Taxonomic identification was performed using the Ribosomal Multilocus Sequence Typing online service (rMLST) (https://pubmlst.org/rmlst/) and searches against PubMLST using the mlst program (version 2.19.0) (https://github.com/tseemann/mlst).

Prokka annotation pipeline (version 1.14.6) was used for prediction and functional annotation of several gene categories in the genome by combining different tools: (1) Ribosomal RNA, with RNAmmer [version 1.2], (2) Carrier RNAs, with ARAGORN [version 1.2.38], (3) Non-coding RNAs, with Infernal [version 1.1.2] and HMMs derived from Rfam [version 14.1], (4) CRISPR clusters, with MinCED (version 0.4.2) [based on the CRT program and available at https://github.com/ctSkennerton/minced], (5) Protein coding genes, predicted with Prodigal (version 2.6.3) and annotated with BLAST + (version 2.9.0) searches against customized versions of ISfinder, NCBI Bacterial Antimicrobial Resistance Reference Gene Database, and UniProtKB [SwissProtKB], (6) Coding genes not annotated in the previous step, annotated with HMMER (version 3.2.1) against the UniprotKB/HAMAP database, (7) signal peptides, identified with SignalP (version 5.0b). To improve annotation, the annotated genome of the closest lineage identified with ReferenceSeeker was added to the database with the “-proteins” parameter of the Prokka pipeline.

For annotating resistance and virulence genes, the draft of the genome was first analyzed with ABRicate version 1.0.1 (https://github.com/tseemann/abricate). This uses BLAST + (version 2.10.0) to search the genome for resistance genes present in cured databases, namely NCBI Bacterial Antimicrobial Resistance Reference Gene Database, CARD, ResFinder, MEGARes, VFDB, and PlasmidFinder. Additionally, AMRFinderPlus (version 3.8.4) was used to search the genome and proteins noted by Prokka, using the NCBI Bacterial Antimicrobial Resistance Reference Gene Database and combining searches with BLAST + and HMMER.

The quality of the assemblies obtained was evaluated using BUSCO (version 4.0.5). The sets of evaluation genes were derived from the OrthoDB database (version 10.0), representing lists of single-copy orthologous genes found in all representatives of certain taxonomic groups. The resulting genome sketch from the assembly with Shovill and the set of proteins noted by Prokka were submitted for evaluation with BUSCO.

The data were tabulated in Microsoft Excel and examined using IBM SPSS Statistics for Windows, Version 21.0 (IBM Corp.) and GraphPad Prism version 7 (GraphPad Software, Inc., San Diego, CA, USA). The frequencies of the different findings were determined, and statistical differences were evaluated for CFUs and relative frequency of isolation. Normality and homogeneity of variance was calculated using the Shapiro-Wilk normality test and F test, respectively. After confirming the non-Gaussian distribution, a test for comparing independent groups was performed (Mann Whitney test).

Among the fecal samples, 29 showed *Enterococcus* growth at the following frequencies: *E. dispar / E. durans / E. hirae* (37.4%)*, E. faecium, E. gallinarum, E. casseliflavu*s (3.45%), and *Enterococcus* not identified at the species level (51.7%). [Figure 1A]. The samples positive for *E. faecium* were from fresh stool. In addition, one sample from dry stool tested positive for *S. aureus*. A greater CFU count was observed in dry droppings than in fresh droppings (*p*<0.05) [Figure 1B]. Species variation was not dependent on the type of sample (dry × fresh; *p*>0.05) [Figure 1C].

**FIGURE 1:**
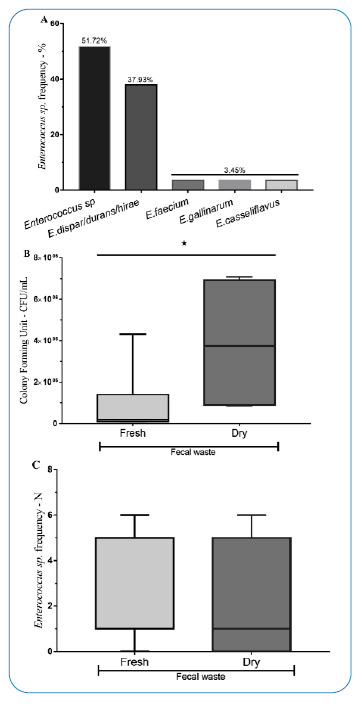
Isolation and identification of bacterial strains in pigeon fecal samples. Relative frequency of isolated *Enterococcus* spp. **(A)** Fecal samples were inoculated on solid medium, and after bacterial growth, the number of colony forming units (CFU) of fresh and dry samples were compared **(B)**. Absolute frequencies of the *Enterococcus sp.* were compared between fresh and dry samples **(C)**. *indicates significant differences (Mann-Whitney test) between the groups, p<0.05.

*S. aureus* was susceptible to all tested antibiotics. In contrast, phenotypic resistance to gentamicin, erythromycin, and ciprofloxacin was observed in *E. faecium*, as well as susceptibility to vancomycin and linezolid.

The metrics for *S. aureus* and *E. faecium* genome assembly, including the total size, N50, and E-size (or auN), are shown in Table 1. Taxonomic identification by rMLST classified one of the isolates as *S. aureus*, with 55 identified alleles, all from *S. aureus*. MLST results indicated that the isolate belonged to *S. aureus* strain type 188 (ST188), with the following alleles: arcC (3), glpF (1), aroE (1), gmk (8), tpi (1), pta (1), and yqiL (1). Moreover, no alleles from other species were identified. No significant contamination was identified using either BlobTools or CheckM (Supplementary Table 1). Among the 1169 *S. aureus* markers present in the CheckM database, 1154 were found in a single copy, 10 were not found, and 5 were duplicates. This indicates good completeness and a low level of contamination.

**TABLE 1:** Metrics of the assembled genome draft of *Enterococcus faecium* and *S. aureus*.

	*E. faecium*	*S. aureus*
Total Length	2864887	2775544
Largest contig	191576	886206
# sequences	120	25
N50	113722	534742
L50	10	2
E-size (auN)	104998	542215
%GC	37.57	32.73

For the *E. faecium* isolate, BlobTools analysis identified a set of contigs as *E. faecium* (contigs adding 2.82 Mb, average coverage of mapping >350 Mb). Taxonomic identification of the draft genome by rMLST confirmed the classification by BlobTools, with the *E. faecium* draft showing 54 alleles. MLST identified the *E. faecium* genome as the type 1054 (ST1054) strain with the following alleles: atpA (9), ddl (3), gdh (1), purK (2), gyd (2), pstS (1), and adk (1). CheckM evaluation of the genomes showed a good degree of completeness and a low rate of contamination (Supplementary Table 1). The Results of BUSCO quality analysis are presented in Supplementary Table 2. A summary of the annotations for Prokka is provided in Table 2 **.** The annotations of antibiotic resistance genes are provided in Supplementary Table 3 for each strain.

Thus, medically important gram-positive cocci were isolated by *C. livia* dropping samples collected from the external environment of hospitals in this study. Compared to other *staphylococci*, *S. aureus* ST188 is highly adapted to different host types. It has shown an increasing prevalence of community-acquired infection on the Asian continent, infecting farm animals, adults, and pediatric patients^8^ ^-^ ^10^. However, we have not found any studies to date that relate *S. aureus* ST188 to the colonization of *C. livia* birds. In connection with healthcare, this strain has been previously described as causing bacteremia in a patient who underwent hospitalization^11^. Despite its phenotypic susceptibility to the tested antibiotics, the isolate demonstrated genetic determinants related to resistance according to molecular analyses. Discrepancies between genotypic and phenotypic results are frequently reported in the literature, and this phenotype/genotype dissociation may be a consequence of genetic alterations promoted by the environment or by other genetic factors; that is, the expression of a resistance phenotype by an agent may be dependent on a certain environmental context and/or on the existence of other genes or mutations^12^. 

**TABLE 2:** Summary of Prokka’s annotation for *E. faecium* and *S. aureus* genomes.

	*E. faecium*	*S. aureus*
tRNAs	56	62
rRNAs	3	9
ncRNAs	58	113
CRISPRs	0	1
CDS	2754	2589

**ncRNA:** non-coding RNA sequences; **rRNA:** ribosomal RNA sequences; **tRNA:** transfer RNA sequences; **CRISPRs:** clusters of regularly interspaced short palindromic repeat sequences; **CDS:** coding sequence.

*Enterococci* are normally present in the intestinal microbiomes of humans and other animals and play a prominent role in hospital environment-related infections^13^. *Enterococci* present an important challenge for antimicrobial therapy owing to their intrinsic resistance to several drugs, as well as their ability to acquire resistance from other bacteria^14^.

Despite presenting susceptibility to vancomycin, *E. faecium* presented phenotypic resistance to gentamicin, erythromycin, and ciprofloxacin and was found to harbor resistance genes for variousantibiotics (Supplementary Table 3). The strain found in this study, *E. faecium* ST1054, was previously identified as a nosocomial agent causing urinary tract infection in a patient admitted to a neurosurgery ward in Portugal^15^.

The presence of domestic pigeons in the external areas of hospitals is a potential risk factor for the transmission of pathogens among patients, especially those undergoing intensive care. Therefore, it is evident that measures should be adopted to control pigeon populations near areas where health facilities are located.

## SUPPLEMENTARY MATERIAL

**SUPPLEMENTARY TABLE 1:** Completeness and contamination analysis of the genome draft made with CheckM.

	# marker	0	1	2	3	4	5+	Completeness	Contamination	H. lineage
*E. faecium*	672	1	669	2	0	0	0	99.61	0.15	0
*S. aureus*	1169	10	1154	5	0	0	0	99.11	0.83	0

## Appendix

**SUPPLEMENTARY TABLE 2:** Analysis of the presence of orthologs in *E. faecium* and *S. aureus* genomes and in the proteins predicted based on these genomes using BUSCO.

BUSCO (n=450)	*E. faecium*	*S. aureus*
Complete, single copy	397 (98.75%)	448 (99.6%)
Complete, duplicated	3 (0.75%)	0 (0.0%)
Fragmented	1 (0.25%)	2 (0.4%)
Missing	1 (0.25%)	0 (0.0%)

## Appendix

**SUPPLEMENTARY TABLE3:** Genotypes and phenotypes associated with resistant strains isolated from C. *livia* near hospitals equipped with intensive care units in Triangulo Mineiro, MG, Brazil. Texts adapted from “Comprehensive Antibiotic Resistance Database” CARD (https://card.mcmaster.ca/home).

Species	Gene	Product	Resistance
*E. faecium*	aac(6')-Ii	Aminoglycoside N-acetyltransferase AAC(6')-Ii	Aminoglycoside
	eat(A)	ABC-F type ribosomal protection protein Eat(A)	Pleuromutilin
	efmA	efmA is an MFS transporter permease in *E. faecium*	Fluoroquinolone, macrolide
	msr(C)	ABC-F type ribosomal protection protein Msr(C)	Macrolide, lincosamide, oxazolidinone, phenicol, pleuromutilin, streptogramin, tetracycline
*S. aureus*	tet(38)	Tetracycline efflux MFS transporter Tet(38)	Tetracycline
	blaZ	Penicillin-hydrolyzing class A beta-lactamase BlaZ	Beta-lactam
	blaR1	Beta-lactam sensor/signal transducer BlaR1	Beta-lactam
	blaI_of_Z	Penicillinase repressor BlaI	Beta-lactam
	ant(4')-Ia	Aminoglycoside O-nucleotidyltransferase ANT(4')-Ia	Kanamycin, tobramycin
	lnu(A)	Lincosamide nucleotidyltransferase Lnu(A)	Lincosamide
	arlR	ArlR is a response regulator that binds to the norA promoter to activate expression. ArlR must first be phosphorylated by ArlS.	Acridine dye, fluoroquinolone
	arlS	ArlS is a protein histidine kinase that phosphorylates ArlR a promoter for norA expression.	Acridine dye, fluoroquinolone
	*Staphylococcus_aureus*_norA	NorA gene cloned from *S. aureus* confers relatively high resistance to hydrophilic quinolones, such as norfloxacin, enoxacin, ofloxacin, and ciprofloxacin, with low or no resistance to hydrophobic quinolones, such as nalidixic acid, oxolinic acid, and sparfloxacin, in *S. aureus* and *E. coli*.	Fluoroquinolone
	mgrA	MgrA, also known as NorR, is a regulator for norA, norB, and tet38. It is a positive regulator for norA expression but is a direct repressor for norB and an indirect repressor of tet38.	Acridine dye, cephalosporin, fluoroquinolone, penam, peptide, tetracycline
	*Staphylococcus_aureus*_LmrS	MFS transporters are secondary active transporters with single-polypeptide chains containing 400-600 amino acids that transport small solutes across the membrane using electrochemical gradients. LmrS has 14 transmembrane helices, and when expressed in *E. coli* is capable of extruding a variety of antibiotics, including linezolid, trimethoprim, florfenicol, chloramphenicol, erythromycin, streptomycin, kanamycin, and fusidic acid.	Aminoglycoside, diaminopyrimidine, macrolide, oxazolidinone, phenicol
	mepR	MepR is an upstream repressor of MepA in *S. aureus*. It is part of the mepRAB operon.	Glycylcycline, tetracycline
	mepA	MepA is an efflux protein regulated by MepR and part of the MepRAB cluster. Its presence in *S. aureus* leads to multidrug resistance, while it has also been shown to decrease tigecycline susceptibility.	Glycylcycline, tetracycline
	ANT(4')-Ib	Kanamycin nucleotidyltransferase sequence from *S. aureus* plasmid. Confers resistance to kanamycin neomycin and other aminoglycosides.	Aminoglycoside
